# Multivariate analysis of febrile neutropenia occurrence in patients with non-Hodgkin lymphoma: data from the INC-EU Prospective Observational European Neutropenia Study

**DOI:** 10.1111/j.1365-2141.2008.07514.x

**Published:** 2008-12-03

**Authors:** Ruth Pettengell, André Bosly, Thomas D Szucs, Christian Jackisch, Robert Leonard, Robert Paridaens, Manuel Constenla, Matthias Schwenkglenks, for the Impact of Neutropenia in Chemotherapy -European Study Group (INC-EU)

**Affiliations:** 1 Cellular and Molecular Medicine, St George’s University of LondonCranmer Terrace, London, UK; 2 Service d’Hématologie, Cliniques Universitaires UCLGodinne, Belgium; 3 European Centre of Pharmaceutical Medicine, University of Basel, c/o ECPM Executive Office, University HospitalBasel, Switzerland; 4 Department of Gynaecology and Obstetrics, Klinikum OffenbachOffenbach, Germany; 5 Cancer Services & Clinical Haematology, Charing Cross HospitalLondon, UK; 6 Department of Medical Oncology, University Hospital GasthuisbergLeuven, Belgium; 7 Servicio de Oncologia, Complexo Hospitalario de PontevedraPontevedra, Spain

**Keywords:** Non-Hodgkin lymphoma, neutropenia, chemotherapy, risk factors

## Abstract

Myelosuppression, particularly febrile neutropenia (FN), are serious dose-limiting toxicities that occur frequently during the first cycle of chemotherapy. Identifying patients most at risk of developing FN might help physicians to target prophylactic treatment with colony-stimulating factor (CSF), in order to decrease the incidence, or duration, of myelosuppression and facilitate delivery of chemotherapy as planned. We present a risk model for FN occurrence in the first cycle of chemotherapy, based on a subgroup of 240 patients with non-Hodgkin lymphoma (NHL) enroled in our European prospective observational study. Eligible patients had an International Prognostic Index of 0–3, and were scheduled to receive a new myelosuppressive chemotherapy regimen with at least four cycles. Clinically relevant factors significantly associated with cycle 1 FN were older age, increasing planned cyclophosphamide dose, a history of previous chemotherapy, a history of recent infection, and low baseline albumin (<35 g/l). Prophylactic CSF use and higher weight were associated with a significant protective effect. The model had high sensitivity (81%) and specificity (80%). Our model, together with treatment guidelines, may rationalise the clinical decision of whether to support patients with CSF primary prophylaxis based on their risk factor profile. Further validation is required.

Chemotherapy-induced neutropenia (CIN) is a frequent and potentially serious adverse effect of cancer treatment (Dale *et al*, 2003). Lymphoma patients with CIN who develop febrile neutropenia (FN) are typically hospitalised and treated with intravenous antibiotics (Dale *et al*, 2003; Crawford *et al*, 2004; Nijhuis *et al*, 2005; Aapro *et al*, 2006; Klastersky & Paesmans, 2007). A common response to CIN is to reduce or delay delivery of chemotherapy treatment (Dale *et al*, 2003; Schwenkglenks *et al*, 2006); however, decreased dose intensity has been associated with increased morbidity and mortality in patients treated with curative intent (Kwak *et al*, 1990; Lepage *et al*, 1993; Bonadonna *et al*, 1995; Budman *et al*, 1998; Bosly *et al*, 2008; Pettengell *et al*, 2008a); indicating that patient outcome is improved when the intensity of chemotherapy treatment is optimal (Bosly *et al*, 2008).

Colony-stimulating factors (CSFs) are used to reduce the risk of developing neutropenic complications and to facilitate delivery of planned chemotherapy dose (Komrokji & Lyman, 2004; Aapro *et al*, 2006; Smith *et al*, 2006). Physicians wishing to identify those patients that should be supported with prophylactic CSF are faced with an array of patient-related and treatment-related factors to consider. Current guidelines recommend CSF support for chemotherapy treatment regimens associated with a high risk of FN (>20%) (Aapro *et al*, 2006; Smith *et al*, 2006). One such regimen is combination chemotherapy with cyclophosphamide, doxorubicin, vincristine and prednisone (CHOP), which has long been the standard treatment for patients with aggressive non-Hodgkin lymphoma (NHL) (Fisher *et al*, 1993). The addition of rituximab to the CHOP regimen (R-CHOP) has further improved patient outcomes (Coiffier *et al*, 2002; Pfreundschuh *et al*, 2006; National Comprehensive Cancer Network Inc, 2008a), making R-CHOP the current standard of care (National Comprehensive Cancer Network Inc, 2008a). CHOP-like chemotherapy carries a significant risk of FN (17–50%) (Morrison *et al*, 2001; Lyman *et al*, 2003; Osby *et al*, 2003; Aapro *et al*, 2006; Bosly *et al*, 2008; Pettengell *et al*, 2008b). In addition to the risk associated with the chemotherapy regimen, other risk factors should be considered in order to determine the patient’s overall FN risk (Aapro *et al*, 2006; Smith *et al*, 2006; National Comprehensive Cancer Network Inc, 2008b).

Several retrospective studies have identified potential risk factors for FN in lymphoma patients, including older age, low baseline blood cell counts, low serum albumin, anaemia, abnormal bone marrow, increased lactate dehydrogenase (LDH), co-morbid renal, cardiovascular or hepatic disease, full or high-risk planned chemotherapy regimen, and lack of CSF prophylaxis (Lyman & Delgado, 2003; Lyman *et al*, 2005; Rabinowitz *et al*, 2006; Teegala *et al*, 2007). However, it is not possible to give a weighting to these risk factors and accurately determine their individual importance. The potential for risk factors identified in retrospective studies to guide targeted CSF use needs to be validated in prospective investigations.

Early, prospective clinical models in lymphoma patients not receiving CSF prophylaxis identified high levels of serum LDH and tumour necrosis factor (TNF) and bone marrow involvement as risk factors for FN (Intragumtornchai *et al*, 2000; Voog *et al*, 2000). Data from several large prospective registries have led to the development of risk models for chemotherapy-induced FN, and the risk factors they have identified are broadly consistent with those highlighted by retrospective studies (Casas *et al*, 2006; Lyman *et al*, 2006; Shayne *et al*, 2007a). However, these studies were in patients with solid tumours (Casas *et al*, 2006) or in patients with solid tumours or lymphoma (Lyman *et al*, 2006; Shayne *et al*, 2007a), and therefore did not specifically examine the risk of FN in lymphoma patients.

Several studies have demonstrated that the risk of FN is greatest in the first cycle of chemotherapy, with >50% of patients who develop FN experiencing an episode during cycle one (Lyman & Delgado, 2003; Lyman *et al*, 2003). The Impact of Neutropenia in Chemotherapy – European Study Group (INC-EU) Prospective Observational European Neutropenia Study was conducted to assess the incidence and predictors of neutropenia, FN and reduced chemotherapy administration for breast cancer and lymphoma patients in European practices. Multivariate regression models for lymphoma patients indicated that first cycle FN, age ≥65 years, disease status, and type of chemotherapy regimen predicted low relative dose intensity (RDI), while primary prophylaxis with CSF was protective (Pettengell *et al*, 2008b).

Here we present a subgroup analysis of NHL patients from the INC-EU prospective study with the aim of establishing a multivariate risk model of FN occurrence in the first cycle of chemotherapy. Such models may help to target high-risk patients for prophylactic treatment in order to decrease the incidence of myelosuppression and enable full-dose chemotherapy to be delivered on schedule.

## Methods

### Study design and patient selection

Data were obtained for 749 patients with histologically confirmed breast cancer, NHL and Hodgkin lymphoma (HL) who were enrolled in the INC-EU Prospective Observational European Neutropenia Study between January 2004 and May 2005. A subset of 240 patients with NHL were included in this sub-analysis. The study was conducted in 66 centres in Belgium, France, Germany, Spain and the UK. Of these, 39 centres contributed NHL patients for this subanalysis. Ethical approval was obtained from the institutional review boards of all centres. Patients with NHL and an International Prognostic Index (IPI) of 0–3, and who were scheduled to receive a new myelosuppressive chemotherapy regimen with at least four cycles, were eligible for inclusion. All participants provided their informed consent. Further details of the overall study design and patient selection have been described previously (Pettengell *et al*, 2008b).

### Statistical methods

Multivariate logistic regression models of FN occurrence in cycle 1 were developed. In line with established definitions (e.g. National Comprehensive Cancer Network Inc, 2008b), FN was defined as Grade 4 CIN [absolute neutrophil count (ANC) <0·5 × 10^9^/l] and a body temperature ≥38°C. General estimating equations (GEE)-based robust standard error (SE) estimates were used to allow for clustering by study centre. The impact of this choice was assessed by comparison with results based on conventional SE estimates.

Candidate predictors were selected based on clinical and statistical grounds (*P*≤0·25 in univariate analysis). To rule out circularity effects, potential direct correlates of the dependent variables of interest were not used. In the model-building process, main effects were identified by manually exploring all plausible combinations of covariates. A model for the occurrence of FN in any cycle of chemotherapy was also developed using similar techniques.

In an effort to make full use of the available information, missing categories were introduced for candidate predictors with more than 5% missing values. Concerns have been raised that this approach can lead to biased estimation results, particularly where covariates have a high proportion of missing values and are strong confounders (Vach & Blettner, 1991; Greenland & Finkle, 1995). Therefore, as an additional sensitivity analysis, alternative models omitting all covariates with more than 5% missing values were estimated and the parameter estimates and standard errors for the remaining covariates were assessed for deviations; these sensitivity analyses did not reveal any relevant distortions.

The Hosmer–Lemeshow goodness of fit test and plots of mean observed *versus* mean predicted event probabilities, by deciles of the linear predictor, were used to assess model fit. The risk of cycle 1 FN is presented as an odds ratio (OR) with 95% confidence interval (CI). Predictive ability of the models was characterised by sensitivity (percentage of the FN occurrences that were correctly predicted) and specificity (percentage of the FN non-occurrences that were correctly predicted), positive predictive value (percentage of patients predicted to have an FN who had FN), negative predictive value (percentage of patients predicted not to have an FN who did not have FN), the area under the receiver operating characteristic (ROC) curve, and the total proportion of correct predictions. Additionally, in the absence of an independent validation dataset, 10-fold cross-validation was performed. In a final step, the model was applied to hypothetical scenarios.

### Variables considered for multivariate models

The following variables were considered for logistic regression model building for both cycle 1 FN and any cycle FN: previous chemotherapy (vs. chemotherapy-naïve); planned doses (for sequential regimens, of first part of chemotherapy); chemotherapy treatment within a clinical trial protocol; CSF prophylaxis (for the purpose of statistical modelling, defined as any CSF use before a FN occurred); antibiotic prophylaxis (for the purpose of statistical modelling, defined as any cotrimoxazole or quinoline use before a FN occurred) cancer stage (Ann Arbor); number of haematology laboratory tests before a grade IV CIN occurred; recent infection (<60 d prior to start to chemotherapy); baseline ANC <3·0 × 10^9^/l; baseline white blood cell count (WBC) <5·0 × 10^9^/l; baseline haemoglobin <100 g/l; baseline glucose >8·8 mmol/l; baseline albumin <35 g/l; baseline total bilirubin >17·1 μmol/l; baseline alkaline phosphatase >250 IU/l; number of comorbidities at baseline; cardiac comorbidity at baseline; vascular comorbidity at baseline; cardiovascular comorbidity at baseline; liver disease at baseline; renal comorbidity at baseline; age; glomerular filtration rate (GFR; estimated using the Cockcroft-Gault formula); height; weight; body surface area (BSA); and body mass index (BMI). Assessment of comorbidities at baseline used Medical Dictionary for Regulatory Activities (MedDRA®)-coded medical history entries with the following system organ class and preferred term names: cardiac disorders; vascular disorders; renal and urinary disorders; hepatobiliary disorders; infections and infestation; diabetes mellitus. For the any cycle model, the following covariates were also considered: planned dose intensities (for sequential regimens, of first part of chemotherapy); use of a dose dense regimen (cycle length 2 weeks instead of 3 weeks); planned cycle length; planned cycle number; dose reduction (≥10% of planned dose of at least one drug in at least one cycle) before FN occurred; and dose delay (a delay ≥4 d in at least one cycle) before FN occurred.

## Results

**Fig 1:**
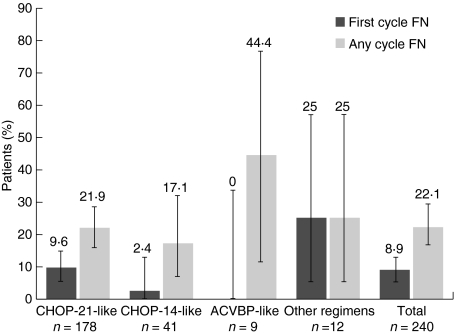
Incidence of febrile neutropenia (FN) in cycle 1 and across all cycles. Error bars represent 95% exact binomial confidence intervals. ACVBP = doxorubicin, cyclophosphamide, vindesine, bleomycin, and prednisone. CHOP = cyclophosphamide, doxorubicin, vincristine and prednisone. Data taken from Pettengell *et al*, 2008b.

Patient and baseline disease characteristics are shown in Table I. The majority of patients (75%) received a CHOP-21-like treatment regimen and a high percentage (82%) of patients received rituximab (Table II). An average of six chemotherapy cycles were planned (mean 6·2, SD 1·5). Overall, 28% of patients received primary CSF prophylaxis and 29% had other CSF use. CSFs used were: filgrastim, 40%; pegfilgrastim, 34%; lenograstim, 10%. The remaining 16% of patients with any CSF use received two or three of these substances. Primary antibiotic prophylaxis with cotrimoxazole was seen in 8% of patients and prophylaxis with quinolones in 14%. During cycle 1, FN occurred in 9% of patients and the incidence of FN across all cycles of chemotherapy was 22% (Fig 1). Grade IV CIN occurred in 35% of patients in cycle 1 and in 54% of patients across all cycles.

**Table I:** Patient and baseline disease characteristics (*n* = 240).

Characteristic	
Age (years), mean ± SD (range)	63·2 ± 12·9 (17–90)
Female gender, *n* (%)	105 (43·8)
Height (cm), mean ± SD (range)	169·9 ± 9 (145–194)
Weight (kg), mean ± SD (range)	75 ± 16 (41–176)
BSA (m^2^), mean ± SD (range)	1·8 ± 0·2 (1·3–2·4)
GFR (ml/min), mean ± SD (range)*	82·9 ± 30·7; (21·6–264·0)
REAL classification, *n* (%)	
Diffuse large cell	154 (64·2)
Follicular	35 (14·6)
Mantle cell	12 (5·0)
Other	36 (15·0)
Unknown	3 (1·3)
Ann Arbor staging, *n* (%)†	
I	42 (17·7)
II	62 (26·2)
III	39 (16·5)
IV	94 (39·7)
B symptoms, *n* (%)†	113 (47·7)
IPI score, *n* (%)†	
Low (0–1)	75 (31·7)
Intermediate (2–3)	132 (55·7)
High (≥4)	30 (12·7)
No. of comorbidities, mean ± SD (range)	2·1 ± 2·1 (0–11)
Cardiovascular comorbidity, *n* (%)	65 (27·1)
Cardiac comorbidity *n* (%)	32 (13·3)
Liver comorbidity *n* (%)	5 (2·1)
Renal comorbidity *n* (%)	16 (6·7)
Recent infection, *n* (%)‡	11 (4·6)
Previous chemotherapy, *n* (%)	25 (10·4)
Low baseline albumin <35 g/dl, *n* (%)§	54 (28·6)
High alkaline phosphatase >250 IU/l, *n* (%)¶	7 (3·1)

BSA, body surface area; GFR, glomerular filtration rate; IPI, International Prognostic Index; REAL, Revised European American Lymphoma; SD, standard deviation.

* *n* = 234.

† *n* = 237.

‡ <60 d prior to start of chemotherapy or ongoing infectious comorbidity.

§ *n* = 189.

¶ *n* = 227.

**Table II:** Treatment characteristics.

Regimen	*n*	Distribution (%)	Primary CSF prophylaxis %‡ (*n*)	Other CSF use* %‡ (*n*)	Rituximab administration %‡ (*n*)
Total	240	100	27·5 (66)	28·8 (69)	81·7 (196)
CHOP-21-like†	178	74·2	19·7 (35)	34·3 (61)	86·5 (154)
CHOP-14-like	41	17·1	75·6 (31)	9·8 (4)	65·9 (27)
ACVBP-like	9	3·8	66·7 (6)	33·3 (3)	77·8 (7)
Other regimens	12	5·0	66·7 (8)	8·3 (1)	66·7 (8)

ACVBP, doxorubicin, cyclophosphamide, vindesine, bleomycin, and prednisone; CHOP, cyclophosphamide, doxorubicin, vincristine and prednisone; CSF, colony-stimulating factor.

* Secondary prophylaxis or treatment.

† Includes six patients with a cycle length of 28 d.

‡ Denominator values for percentage calculations are the regimen *n*-values in column 2.

The results of the multivariate logistic regression analysis used to model risk factors for cycle 1 FN are shown in Table III. Clinically relevant factors that were significantly associated with cycle 1 FN were older age, increasing planned cyclophosphamide dose, increasing planned etoposide dose, a history of previous chemotherapy, a history of recent infection, and low baseline albumin <35 g/l. Prophylactic CSF use and higher weight were associated with a significant protective effect. The effect of antibiotic prophylaxis with cotrimoxazole or quinolones remained non-significant [OR (95% CI): 0·36 (0·08–1·62), *P* = 0·181] when added to the final model. Replacing age with GFR (to which it is inversely related) and replacing weight with height yielded similar models.

**Table III:** Logistic regression model for predicting cycle 1 FN occurrence*.

Variable	Odds ratio	95% CI	*P*-value
Age†	2·20	1·21–4·01	0·010
Weight‡	0·62	0·43–0·89	0·010
Previous chemotherapy§	6·39	1·72–23·68	0·006
Planned cyclophosphamide dose§	1·16	1·02–1·32	0·023
Planned cytarabine dose§	1·06	0·98–1·16	0·151
Planned etoposide dose§	1·59	1·20–2·11	0·001
CSF before an event occurred¶	0·18	0·03–0·94	0·042
Baseline albumin low**	4·76	1·35–16·71	0·015
Baseline albumin missing**	0·52	0·09–2·99	0·464
Recent infection††	3·07	0·99–9·52	0·052

CI, confidence interval; CSF, colony-stimulating factor.

* Number of observations = 240, Wald χ^2^ = 26·59, prob > χ^2^ = 0·003, log pseudolikelihood = −52·41.

† Per additional 10 years of age.

‡ Per additional 10 kg body weight.

§ Planned doses in mg/m^2^ body surface area; per additional 50 mg/m^2^.

¶ Myelopoietic growth factor use before a FN occurred in cycle 1.

** Baseline albumin <35 g/dl, missing category introduced to avoid loss of observations (sensitivity analyses did not reveal any relevant distortions with the use of this technique).

†† During 60 d prior to chemotherapy or ongoing infectious comorbidity.

**Fig 2:**
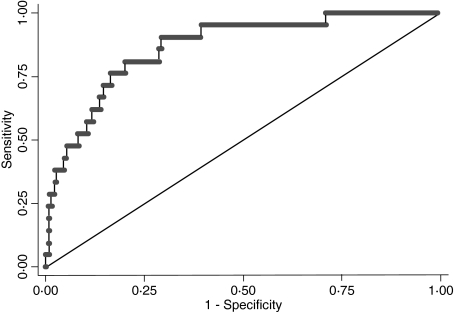
Receiver operating characteristic (ROC) curve for the multivariate analysis of factors predicting cycle 1 febrile neutropenia. Area under ROC curve = 0·86 (95% confidence interval 0·79–0·94).

The model correctly classified 192 of the 240 patients (80%). The area under the ROC curve, which describes the ability of the model to discriminate between those at risk from cycle 1 FN and those not at risk, was 0·86 (95% CI 0·79–0·94) (Fig 2). (An area under the ROC curve of 0·5 implies an ability to discriminate that is no better than chance, while a value of 1 represents perfect ability to discriminate). When the optimal probability cut-off was used to predict cycle 1 FN, test characteristics were: sensitivity 81%; specificity 80%; positive predictive value 28% (proportion of patients classified as high risk who suffered cycle 1 FN); negative predictive value 98% (the proportion of patients classified as low FN risk who did not suffer cycle 1 FN). Predictive ability was only slightly lower under 10-fold cross-validation conditions (area under the ROC curve 0·78).

A similar model was developed to predict the risk of FN in any cycle. In agreement with the first cycle FN model, the following factors were also significantly associated with FN occurrence in any cycle: age [OR (95% CI): 1·79 (1·16–2·78) per additional 10 years, *P* = 0·009]; increasing planned cyclophosphamide dose [OR (95% CI): 1·33 (1·16–1·52) per additional 50 mg/m^2^, *P*< 0·001]; increasing planned etoposide dose [OR (95% CI): 1·88 (1·10–3·20) per additional 50 mg/m^2^, *P* = 0·021]; and recent infection [OR (95% CI): 3·32 (1·03–10·71), *P* = 0·044]. Likewise, prophylactic CSF use [OR (95% CI): 0·21 (0·10–0·44), *P*< 0·001] and higher weight [OR (95% CI): 0·62 (0·44–0·88) per additional 10 kg, *P* = 0·007] were associated with a significant protective effect. In addition, the following clinically relevant factors were associated with a significantly increased risk of any cycle FN: low baseline ANC or WBC [ANC <3·0 × 10^9^/l or WBC <5 × 0^9^/l; OR (95% CI): 4·18 (1·82–9·60), *P* = 0·001], high baseline alkaline phosphatase [>250 IU/ml; OR (95% CI): 9·07 (1·41–58·50), *P* = 0·020], cardiovascular comorbidity [OR (95% CI): 2·56 (1·04–6·29), *P* = 0·041], and increasing planned cytarabine dose [OR (95% CI): 1·09 (1·05–1·13) per additional 50 mg/m^2^, *P*< 0·001]. Use of a dose dense regimen (cycle length 2 weeks instead of 3 weeks) may influence FN but did not attain statistical significance in our model [OR (95% CI): 1·84 (0·71–78), *P* = 0·208; see Discussion]. In the any cycle model, dose reductions before an FN event occurred [OR (95% CI): 0·24 (0·09–0·63), *P* = 0·004] and dose delays before an FN event occurred [OR (95% CI): 0·17 (0·07–0·40), *P*< 0·001] had a significant protective effect against FN. In contrast to the cycle 1 model, a history of chemotherapy [OR (95% CI): 1·76 (0·49–6·36), *P* = 0·390] and low baseline albumin [OR (95% CI): 1·62 (0·54–4·85) *P* = 0·391 when added to the final model] were non-significant in the any cycle model. Antibiotic prophylaxis showed no effect.

The any cycle model correctly classified 180 of 237 patients (76%). The area under the ROC curve was 0·83 (95% CI 0·76–0·90). When the optimal probability cut-off was used to predict any cycle FN, test characteristics were: sensitivity 76%; specificity 76%; positive predictive value 48%; negative predictive value 92%. Predictive ability was slightly lower under 10-fold cross-validation conditions (area under the ROC curve 0·72).

Based on our models, the estimated risk of FN in cycle 1 or any cycle during R-CHOP therapy for lymphoma (without CSF prophylaxis) in a hypothetical 80 kg subject (average weight of our male subsample) is shown in Table IV. The risk of FN increased as the number of risk factors and age increased in both models. Assigning a lower weight (e.g. 55 kg) to the subject increased the risk for all possible scenarios shown in Table IV.

**Table IV:** Estimated risk [%] of cycle 1 FN and any cycle FN following R-CHOP treatment for NHL (cycle length 3 weeks) in an 80 kg subject (average weight of male subsample) according to age and risk factor profile. Estimated risks for a lower assigned weight (55 kg) are given in parentheses.

	Age, years; weight 80 kg (55 kg), [%]				
	
Cycle/risk factors	35	45	55	65	75
*Cycle 1*					
None	0 (1)	1 (2)	1 (4)	3 (8)	6 (16)
Previous CT	2 (5)	3 (10)	7 (21)	15 (36)	28 (55)
Low albumin*	1 (4)	3 (8)	6 (16)	11 (30)	22 (48)
Recent infection†	1 (2)	2 (5)	4 (11)	8 (21)	16 (37)
Previous CT + low albumin*	7 (20)	15 (36)	27 (55)	45 (73)	64 (86)
Previous CT + low albumin* + recent infection†	19 (44)	34 (63)	54 (79)	72 (89)	85 (95)
*Any cycle*					
None	5 (14)	8 (23)	14 (35)	22 (49)	34 (63)
Previous CT	8 (22)	14 (34)	22 (48)	34 (62)	48 (75)
ANC/WBC low‡	17 (41)	27 (55)	40 (69)	55 (80)	68 (88)
Alkaline phosphatase high‡	31 (60)	45 (73)	59 (83)	72 (90)	82 (94)
CV comorbidity	11 (30)	19 (43)	29 (57)	42 (71)	57 (81)
Recent infection†	14 (35)	23 (49)	35 (64)	49 (76)	63 (85)
ANC/WBC low‡ + CV comorbidity	35 (64)	49 (76)	63 (85)	75 (91)	85 (95)
ANC/WBC low‡ + CV comorbidity + recent infection†	64 (85)	76 (91)	85 (95)	91 (97)	95 (98)

ANC, absolute neutrophil count; CT, chemotherapy; CV, cardiovascular; WBC, white blood cell count.

* Baseline albumin <35 g/l.

† During 60 d prior to treatment.

‡ Baseline ANC <3·0 × 10^9^/l or WBC <5·0 × 10^9^/l; baseline alkaline phosphatase >250 IU/ml.

## Discussion

This study identified several clinically relevant factors that were predictive or protective for cycle 1 FN. Patient and baseline characteristics of older age and low baseline albumin were predictive of cycle 1 FN, as were a clinical history of previous chemotherapy or recent infection. Treatment characteristics, specifically increasing planned chemotherapy dose, also significantly increased risk of cycle 1 FN. In contrast, higher weight and prophylactic CSF use were associated with significant protective effects.

Older age (>65 years) is recognised as a risk factor for FN by current European guidelines (Aapro *et al*, 2006). Indeed, European Organisation for Research and Treatment of Cancer (EORTC) Elderly Guidelines recommend primary prophylaxis with CSF for all elderly patients receiving curative CHOP-like chemotherapy (Repetto *et al*, 2003). Although many of the other patient risk factors identified in this study do not necessarily reflect risk factors highlighted in the guidelines, it is important to recognise that the EORTC guidelines (Aapro *et al*, 2006) are based on a literature review of studies across tumour types and are not specific for NHL.

The increased risk for cycle 1 FN associated with age and low baseline albumin, and the protective effects of CSF prophylaxis, are consistent with data from retrospective studies specific to NHL patients (Lyman & Delgado, 2003; Rabinowitz *et al*, 2006; Teegala *et al*, 2007). An increased risk of FN in patients with low serum albumin (Intragumtornchai *et al*, 2000) or higher cyclophosphamide dose (Voog *et al*, 2000) was also reported in early prospective studies in this patient population. Data on the potential relationship between prior chemotherapy, weight or recent infection and the risk of cycle 1 FN in NHL is limited. However, a US nationwide prospective cohort study of 3468 patients with solid tumours or lymphoma identified prior chemotherapy and concurrent antibiotics as risk factors for neutropenic complications in cycle 1. We assume that antibiotics are not in themselves a risk factor for FN, but that they are prescribed to patients who are perceived to be at higher FN risk. Other risk factors identified in the US study were the number of myelosuppressive agents, anthracycline-based regimens, planned chemotherapy delivery >85% of standard, cancer type, phenothiazines, abnormal alkaline phosphatase, elevated bilirubin, low platelets, elevated glucose and reduced GFR, whereas CSF prophylaxis was protective (Lyman *et al*, 2006). Results from a subset of older patients from the same registry (*n* = 1378) supported some of these findings and additionally highlighted chemotherapy regimens containing cyclophosphamide, etoposide or ifosfamide as increasing the risk of early neutropenic events (Shayne *et al*, 2007a). Overall, the findings of the US prospective study (Lyman *et al*, 2006; Shayne *et al*, 2007a) and the present study were generally consistent and differences observed may be related to the patient populations studied, treatment regimens and sample size.

It is noteworthy that increasing planned chemotherapy dose was predictive of FN in our model, in keeping with a previously published model (Voog *et al*, 2000) and a recent validated risk model that found that regimens containing cyclophosphamide, etoposide or ifosfamide were associated with an increased risk of early neutropenic events (Shayne *et al*, 2007a). In our model, planned cyclophosphamide use also correlated with the use of other anti-malignant agents, which could potentially mask the contribution of these other agents to the neutropenic potential of the chemotherapy regimen. Planned etoposide dose was identified as a significant predictor of cycle 1 FN; however, very few patients received this agent. Similarly, risk estimates for recent infection were based on a small number of observations (11 patients) and require careful interpretation. CSF primary prophylaxis had a significant protective effect against cycle 1 FN. The protective effects of CSF have been validated previously (Komrokji & Lyman, 2004; Aapro *et al*, 2006; Smith *et al*, 2006).

The high number of patients correctly classified by the model (80%) suggests that it may have potential clinical utility. The model showed good ability to discriminate between patients at risk from cycle 1 FN and those not at risk. Model test characteristics were comparable to, or better than, values published for other risk models of neutropenia (Morrison *et al*, 2001; Lyman & Delgado, 2003; Lyman *et al*, 2006; Rabinowitz *et al*, 2006; Shayne *et al*, 2007a,b). The 98% negative predictive value showed that the model successfully identified patients at low risk of developing FN. The 28% positive predictive value (PPV) indicated that the model identified as high risk some patients who did not ultimately have a cycle 1 FN event. While a higher PPV is desirable, it should be remembered that the PPV in this setting was partially driven by a low absolute frequency of cycle 1 FN events and that not every patient who is at high risk of FN will actually experience FN.

The potential clinical utility of the model was explored by applying our dataset to hypothetical scenarios of NHL patients and estimating the risk of developing FN in cycle 1 or any cycle. Whilst the presence of some risk factors alone (e.g. low baseline albumin) did not predict a high risk of FN, a combination of risk factors increased the predicted risk for developing cycle 1 FN substantially. In addition, patient characteristics of older age or lower weight increased the predicted risk for developing cycle 1 FN for any of the given risk factor scenarios.

Owing to the sample size, the relatively infrequent occurrence of cycle 1 FN (9%) and the high number of covariates used, the logistic regression model generated has some potential limitations in its ability to correctly assess the impact of rare risk factors and there is the possibility of artefacts. This caveat particularly applies to the effects of some comorbidities and baseline laboratory abnormalities. The standard approach to randomly split the study dataset into a training dataset (on which the model is estimated) and a test dataset (on which the model is validated) was considered to be inefficient for the same reasons. Ten-fold cross-validation has been shown to be superior in small datasets (Goutte, 1997) and showed favourable results in the present case. However, additional validation in an independent data set from a different population is clearly required.

The any cycle model correctly classified 76% of patients, and the test characteristics were comparable to a recent model of risk for severe or febrile neutropenia across four cycles of chemotherapy (Shayne *et al*, 2007b). The findings of the any cycle model were similar to those observed with the cycle 1 model, with older age and increasing chemotherapy dose identified as clinically relevant predictors of FN and prophylactic CSF use and higher weight identified as being protective. Although the use of a dose-dense regimen appeared predictive of FN it did not attain statistical significance, which is probably because most patients (76%) treated with dose-dense regimens received CSF support. In addition, dose reductions and dose delays before an event occurred had a significant protective effect against FN. However, the practice of reducing or delaying chemotherapy treatment in response to CIN and FN has been questioned (Dale *et al*, 2003; Schwenkglenks *et al*, 2006), as decreased dose intensity has been associated with increased morbidity and mortality (Kwak *et al*, 1990; Lepage *et al*, 1993; Bonadonna *et al*, 1995; Budman *et al*, 1998; Bosly *et al*, 2008; Pettengell *et al*, 2008a). Cardiac comorbidity was also identified as a risk factor for any cycle FN, which is consistent with current treatment guidelines (Aapro *et al*, 2006).

In summary, this study describes a prospective multivariate risk model that was able to identify clinically relevant factors that were predictive or protective of cycle 1 FN and correctly identify a high proportion of patients at risk of first cycle FN. Our model, together with treatment guidelines, may rationalise the clinical decision of whether to support patients with CSF primary prophylaxis based on their risk factor profile. Further validation is required.

## References

[b1] Aapro MS, Cameron DA, Pettengell R, Bohlius J, Crawford J, Ellis M, Kearney N, Lyman GH, Tjan-Heijnen VC, Walewski J, Weber DC, Zielinski C (2006). EORTC guidelines for the use of granulocyte-colony stimulating factor to reduce the incidence of chemotherapy-induced febrile neutropenia in adult patients with lymphomas and solid tumours. European Journal of Cancer.

[b2] Bonadonna G, Valagussa P, Moliterni A, Zambetti M, Brambilla C (1995). Adjuvant cyclophosphamide, methotrexate, and fluorouracil in node-positive breast cancer: the results of 20 years of follow-up. New England Journal of Medicine.

[b3] Bosly A, Bron D, Van HA, De BR, Berneman Z, Ferrant A, Kaufman L, Dauwe M, Verhoef G (2008). Achievement of optimal average relative dose intensity and correlation with survival in diffuse large B-cell lymphoma patients treated with CHOP. Annals of Hematology.

[b4] Budman DR, Berry DA, Cirrincione CT, Henderson IC, Wood WC, Weiss RB, Ferree CR, Muss HB, Green MR, Norton L, Frei E (1998). Dose and dose intensity as determinants of outcome in the adjuvant treatment of breast cancer. The Cancer and Leukemia Group B. Journal of the National Cancer Institute.

[b5] Casas AM, Rifä J, Gonzalez Larriba JL, Carrato A, Lopez Pousa A (2006). Risk assessment model for haematologic toxicity (HT) in patients with solid tumours (ST) during the first chemotherapy (CT) cycle. Journal of Clinical Oncology, 2006 ASCO Annual Meeting Proceedings Part 1.

[b6] Coiffier B, Lepage E, Briere J, Herbrecht R, Tilly H, Bouabdallah R, Morel P, Van Den Neste E, Salles G, Gaulard P, Reyes F, Gisselbrecht C (2002). CHOP chemotherapy plus rituximab compared with CHOP alone in elderly patients with diffuse large-B-cell lymphoma. New England Journal of Medicine.

[b7] Crawford J, Dale DC, Lyman GH (2004). Chemotherapy-induced neutropenia: risks, consequences, and new directions for its management. Cancer.

[b8] Dale DC, McCarter GC, Crawford J, Lyman GH (2003). Myelotoxicity and dose intensity of chemotherapy: reporting practices from randomized clinical trials. Journal of the National Comprehensive Cancer Network.

[b9] Fisher RI, Gaynor ER, Dahlberg S, Oken MM, Grogan TM, Mize EM, Glick JH, Coltman CA, Miller TP (1993). Comparison of a standard regimen (CHOP) with three intensive chemotherapy regimens for advanced non-Hodgkin’s lymphoma. New England Journal of Medicine.

[b10] Goutte C (1997). Note on free lunches and cross-validation. Neural Computation.

[b11] Greenland S, Finkle WD (1995). A critical look at methods for handling missing covariates in epidemiologic regression analyses. American Journal of Epidemiology.

[b12] Intragumtornchai T, Sutheesophon J, Sutcharitchan P, Swasdikul D (2000). A predictive model for life-threatening neutropenia and febrile neutropenia after the first course of CHOP chemotherapy in patients with aggressive non-Hodgkin’s lymphoma. Leukemia & Lymphoma.

[b13] Klastersky J, Paesmans M (2007). Risk-adapted strategy for the management of febrile neutropenia in cancer patients. Supportive Care in Cancer.

[b14] Komrokji RS, Lyman GH (2004). The colony-stimulating factors: use to prevent and treat neutropenia and its complications. Expert Opinion on Biological Therapy.

[b15] Kwak LW, Halpern J, Olshen RA, Horning SJ (1990). Prognostic significance of actual dose intensity in diffuse large-cell lymphoma: results of a tree-structured survival analysis. Journal of Clinical Oncology.

[b16] Lepage E, Gisselbrecht C, Haioun C, Sebban C, Tilly H, Bosly A, Morel P, Herbrecht R, Reyes F, Coiffier B (1993). Prognostic significance of received relative dose intensity in non-Hodgkin’s lymphoma patients: application to LNH-87 protocol. Annals of Oncology.

[b17] Lyman GH, Delgado DJ (2003). Risk and timing of hospitalization for febrile neutropenia in patients receiving CHOP, CHOP-R, or CNOP chemotherapy for intermediate-grade non-Hodgkin lymphoma. Cancer.

[b18] Lyman GH, Morrison VA, Dale DC, Crawford J, Delgado DJ, Fridman M (2003). Risk of febrile neutropenia among patients with intermediate-grade non-Hodgkin’s lymphoma receiving CHOP chemotherapy. Leukemia & Lymphoma.

[b19] Lyman GH, Lyman CH, Agboola O (2005). Risk models for predicting chemotherapy-induced neutropenia. Oncologist.

[b20] Lyman GH, Kuderer NM, Crawford J, Wolff DA, Culakova E, Poniewierksi MS, Dale DC (2006). Prospective validation of a risk model for first cycle neutropenic complications in patients receiving cancer chemotherapy. Journal of Clinical Oncology.

[b21] Morrison VA, Picozzi V, Scott S, Pohlman B, Dickman E, Lee M, Lawless G, Kerr R, Caggiano V, Delgado D, Fridman M, Ford J, Carter WB (2001). The impact of age on delivered dose intensity and hospitalizations for febrile neutropenia in patients with intermediate-grade non-Hodgkin’s lymphoma receiving initial CHOP chemotherapy: a risk factor analysis. Clinical Lymphoma.

[b22] National Comprehensive Cancer Network Inc (2008a). NCCN Clinical Practice Guidelines in Oncology - Non-Hodgkin’s Lymphomas V.3.2008.

[b23] National Comprehensive Cancer Network Inc (2008b). NCCN Clinical Practice Guidelines in Oncology- Prevention and Treatment of Cancer-Related Infections V.1.2008.

[b24] Nijhuis CO, Kamps WA, Daenen SM, Gietema JA, van der Graaf WT, Groen HJ, Vellenga E, Ten Vergert EM, Vermeulen KM, de Vries-Hospers HG, de Bont ES (2005). Feasibility of withholding antibiotics in selected febrile neutropenic cancer patients. Journal of Clinical Oncology.

[b25] Osby E, Hagberg H, Kvaloy S, Teerenhovi L, Anderson H, Cavallin-Stahl E, Holte H, Myhre J, Pertovaara H, Bjorkholm M (2003). CHOP is superior to CNOP in elderly patients with aggressive lymphoma while outcome is unaffected by filgrastim treatment: results of a Nordic Lymphoma Group randomized trial. Blood.

[b26] Pettengell R, Schwenkglenks M, Bosly A (2008a). Association of reduced relative dose intensity and survival in lymphoma patients receiving CHOP-21 chemotherapy. Annals of Hematology.

[b27] Pettengell R, Schwenkglenks M, Leonard R, Bosly A, Paridaens R, Constenla M, Szucs TD, Jackisch C (2008b). Neutropenia occurrence and predictors of reduced chemotherapy delivery: results from the INC-EU prospective observational European neutropenia study. Supportive Care in Cancer.

[b28] Pfreundschuh M, Trumper L, Osterborg A, Pettengell R, Trneny M, Imrie K, Ma D, Gill D, Walewski J, Zinzani PL, Stahel R, Kvaloy S, Shpilberg O, Jaeger U, Hansen M, Lehtinen T, Lopez-Guillermo A, Corrado C, Scheliga A, Milpied N, Mendila M, Rashford M, Kuhnt E, Loeffler M (2006). CHOP-like chemotherapy plus rituximab versus CHOP-like chemotherapy alone in young patients with good-prognosis diffuse large-B-cell lymphoma: a randomised controlled trial by the MabThera International Trial (MInT) Group. Lancet Oncology.

[b29] Rabinowitz AP, Weiner NJ, Tronic BS, Fridman M, Liberman RF, Delgado DJ (2006). Severe neutropenia in CHOP occurs most frequently in cycle 1: a predictive model. Leukemia & Lymphoma.

[b30] Repetto L, Biganzoli L, Koehne CH, Luebbe AS, Soubeyran P, Tjan-Heijnen VC, Aapro MS (2003). EORTC Cancer in the Elderly Task Force guidelines for the use of colony-stimulating factors in elderly patients with cancer. European Journal of Cancer.

[b31] Schwenkglenks M, Jackisch C, Constenla M, Kerger JN, Paridaens R, Auerbach L, Bosly A, Pettengell R, Szucs TD, Leonard R (2006). Neutropenic event risk and impaired chemotherapy delivery in six European audits of breast cancer treatment. Supportive Care in Cancer.

[b32] Shayne M, Culakova E, Dale DC, Poniewierksi MS, Wolff DA, Crawford J, Lyman G (2007a). A validated risk model for early neutropenic events in older cancer patients receiving systemic chemotherapy. Journal of Clinical Oncology, 2007 ASCO Annual Meeting Proceedings Part 1.

[b33] Shayne M, Culakova E, Poniewierksi MS, Wolff DA, Dale DC, Crawford J, Lyman GH (2007b). Dose intensity and hematologic toxicity in older cancer patients receiving systemic chemotherapy. Cancer.

[b34] Smith TJ, Khatcheressian J, Lyman GH, Ozer H, Armitage JO, Balducci L, Bennett CL, Cantor SB, Crawford J, Cross SJ, Demetri G, Desch CE, Pizzo PA, Schiffer CA, Schwartzberg L, Somerfield MR, Somlo G, Wade JC, Wade JL, Winn RJ, Wozniak AJ, Wolff AC (2006). 2006 update of recommendations for the use of white blood cell growth factors: an evidence-based clinical practice guideline. Journal of Clinical Oncology.

[b35] Teegala SR, Zhou X, Huen A, Ji Y, Fayad LE, Romaguera JE, Vadhan-Raj S (2007). Risks factors for neutropenic fever in lymphoma patients receiving chemotherapy. Journal of Clinical Oncology, 2007 ASCO Annual Meeting Proceedings Part 1.

[b36] Vach W, Blettner M (1991). Biased estimation of the odds ratio in case-control studies due to the use of ad hoc methods of correcting for missing values for confounding variables. American Journal of Epidemiology.

[b37] Voog E, Bienvenu J, Warzocha K, Moullet I, Dumontet C, Thieblemont C, Monneret G, Gutowski MC, Coiffier B, Salles G (2000). Factors that predict chemotherapy-induced myelosuppression in lymphoma patients: role of the tumor necrosis factor ligand-receptor system. Journal of Clinical Oncology.

